# Supplementary material for: Patterns of Intron Gain and Loss in Fungi
Source: PLoS Biol. 2004 Nov 30;2(12):e422. doi: 10.1371/journal.pbio.0020422 (PMC532390; doi:10.1371/journal.pbio.0020422)
Supplement: Table S1 — Also available at http://genes.mit.edu/NielsenEtAl/. (4.3 MB ZIP). [file pbio.0020422.st001.zip › NielsenEtAl/html/789.html]

AN8056.1.NCU06216.1.MG08841.1.FG02510.1


```
 CLUSTAL W (1.82) Multiple Sequence Alignments - Introns Inserted


Sequence 1: NCU06216.1	1262 aa
Sequence 2: MG08841.1	1036 aa
Sequence 3: FG02510.1	1021 aa
Sequence 4: AN8056.1	1114 aa
Alignment Length: 1362 aa
Number Identitical Residues: 419 aa
Alignment Score (without introns) 22007


MG08841.1 	MPARASTRSTRTSGVSHKSSSATLNPRASHASRVSTTSIDVPEETPDNALRHQIAAVFRD
NCU06216.1	MAPGRSTRATRG-------------------ARQTSVVPEAPLDTPDNALRTQVATVFGD
FG02510.1 	MPARTATRSSRTS-------------VARSSVASRASSVPEAVDTPDNALRTKVCAIFRD
AN8056.1  	MPSRVSARSTSTASRKGSTQTATSG-RAGSATPSFAIPEEPALPEAVPTLRRDVCAIFAD
          	*..  ::*::  :. . .:.::: .  :  :    :     .   .  :** .:.::* *

MG08841.1 	AQRTTAAHRKLAVTLRKIQEACCYEP-TSSKT-SAASD--------FGEDDFNAEVARCV
NCU06216.1	AQKTTASHRKLAVTLRKIQEACCYEP-TSTKKPAEADE--------FEEDDFNKEFVRCV
FG02510.1 	AQRTTATHRKLVVNLRKIHESCAYEH-TNPDP-SQVDE--------FDEDAFNREFIRCV
AN8056.1  	AQRSTAGHRKLVVRLRKIQEVCCAIPQKNSKKDSSTEERLIPGEETVPEKEFNVEVGRCV
          	**::** ****.* ****:* *.   .....  : ..:   ....:. *. ** *. ***

MG08841.1 	LRIMPVKKTESAGEKVVKFIAFFLPHAIVKDNELLG--EVDEDASTMPETPTTRLMGHLM
NCU06216.1	LRVMPIKKSEGVGEKTVRFIGLFLRHAVEKDNEILG--EADPDASTMPETPSTRLTGHLM
FG02510.1 	GKIMPIKKSESVGEKSIRFTGLFLQHASAKDNELLG--DIDEDASVMPETPSTRLIAQLL
AN8056.1  	LRILSIKKTEPVGDRILRFLGNFLTHASEKDAEIFGSEEDEDDMQNSHERPTAHLTTSLV
          	 :::.:**:* .*:: ::* . ** **  ** *::*:.: : * .   * *:::*   *:

MG08841.1 	DTILPLLSAKDKYVRYRSTQLVSQIINSLDAIDDDLFQKLRYGLLKRIRDKEAMVRAQAV
NCU06216.1	ETVLPLMIAKDKFVRYRSTQLISHIINSLDAIDDDLFQKLRSSLLRRIRDKEAMVRAQAV
FG02510.1 	EAILPLLTAKDKVVRFRSTQLISHIINSLDAIDDDLFQKLRHGLLKRIRDKEAMVRSQAV
AN8056.1  	SLLVPLLSAKDKVVRFRTTQIIAHIVNSLDTVDDELYHTLRQGLLKRIRDKEPSVRVQAV
          	. ::**: **** **:*:**::::*:****::**:*::.** .**:******. ** ***

MG08841.1 	LGLGRLAGNQMEAEPNSDDSDEDASTELLPKLLEVLQNDPSADVRRSLLVNLPILPNTLP
NCU06216.1	LGLGRLAGNQFEGEPNSDDSDDDGSTGLLEKLLEVLQNDPSADVRRSLLVNLPILPTTLP
FG02510.1 	LGLGRLAGNQTEGCTNSDDSDDD-DTGLLEKLLEVLQHDPNADVRRSLLVNLPILPETLS
AN8056.1  	MGLGRLAGNEE------DDDENDDTSALVEKLVDIMQNDTAAEVRRTLLLNLPLIPSTLP
          	:********:       **.::*  : *: **::::*:*. *:***:**:***::* **.

MG08841.1 	FLLERARDSDALTRRAVYSRLLPALGDFRHLSLSMREKLLRWGLRDRDENVRKAAGRLFR
NCU06216.1	YLLERARDQDALTRRAVYSRLLPALGDFRHLSLSMREKLLRWGLRDRDENVRKAACRLFR
FG02510.1 	VLLERARDQDAATRRAVYSRLLPALGDFRHLSVKMREKLLRWGIRDRDENVRKAASRLFR
AN8056.1  	YLLERARDLDAPTRRALYSRLLPTLGDFRHLSLSMREKLLRWGLRDRDESVRKATGKLFY
          	 ******* ** ****:******:********:.*********:*****.****: :** 

MG08841.1 	ERWIEDCVGPPEETEEGQPAQPAAPNFDGLLELLERIDVVNSGVENGVALEAMKGFWEGR
NCU06216.1	ERWIEDCAGTAPPVEGGQPSEVSPPSIEALLELLERIDVVNSGGENGVALEAMKGFWEGR
FG02510.1 	ERWIEDCAG-APPQEDGTAAEPAPPNFDGLCELLERIDVVNTGVENGVALEAMKGFWEGR
AN8056.1  	DRWIEDIAGTNNDPE--KFGQRSEPRIPALLELLERIDVVNSGMESGIAHEAMRSFWEGR
          	:***** .*.    *    .: : * : .* **********:* *.*:* ***:.*****

MG08841.1 	PDYRDAACFDDNFWETLSAESVFMARSFNQFCRTEGNGRYESLVEEKLPEVTKLAFYLER
NCU06216.1	ADYRNAVNFDDNFWETLSSESVFVARTFNDFCRNEGNGKYESLVDEKLPEVTKLAFYLER
FG02510.1 	PDYRETVEFDDNFWETLSAESVFMARSFNEFCRLEGNGKYLNLLEEKLPEVTKLAFFLER
AN8056.1  	PDYREAVLFDEAFWESMTAESAFLLRSFNDFCRVENEGKYDSLADEKIPVVTALAMYLHK
          	.***::. **: ***::::**.*: *:**:*** *.:*:* .* :**:* ** **::*.:

MG08841.1 	YIKVLIDAIKRVEEQEVGEDEDEEDTVEQEFIVEQLLQIALTLDYSDEVGRRKMFALLRQ
NCU06216.1	YVKVLIDAIKRINEQEVEDEEEEEDTVEQEFIVEQLLHIALTLDYSDEVGRRKMFALLRQ
FG02510.1 	YLHVLVAAVKRVA--EADEDDEEEDTVEQEFIVEQMLQIALTLDYSDEVGRRKMFTLLRQ
AN8056.1  	YMTELLQRKKLTKD---ATDVNDDDTVEIEFIVEQLLHIAMTLDYSDEVGRRKMFSLLRE
          	*:  *:   *   .     : :::**** ******:*:**:**************:***:

MG08841.1 	TLSIPELPDEVTKLTVDVLRDICAPDAAGEREFCSVVLEAVADVHDTIVDDMGG----DE
NCU06216.1	TLSIPELPDEVTKLTVQVLRDLCAPDAAGEKEFCSIVLEAVADVHDTIVDDVPDEGDIDD
FG02510.1 	AISIPDLPDEVTKLAVEVLQGICSPDAAGEREFISIVLESVADVHDTIVDDPPPD---DA
AN8056.1  	ALAVPELPQESTKLAVETLRCVCGPDAAAESEFCSVVLEAIAEVHDTIS----------T
          	::::*:**:* ***:*:.*: :*.****.* ** *:***::*:*****            

MG08841.1 	DESFHSARSEVSSDGTPTKGGKPDKDAN---LSEEEKQEKLIKELMINLKCLHIVQCMLA
NCU06216.1	NESFHSARSEVSDDEDSQSKKKKNKGPE---LSEEEAAQKAIKEIMINMKCLHIVQCMLT
FG02510.1 	EDSFHSARSEVSGPSTPTKS-KRGGSPE---ISEEEAQEKAVREIVVNMKCLHIVQCMLT
AN8056.1  	EDSFVSAKSEISDDASSRQRSETPMSEDDKPFNKEEAKAKVLKEIVINMKCLHIALCMLQ
          	::** **:**:*.   . .  :   . :...:.:**   * ::*:::*:*****. *** 

MG08841.1 	NVSGSLQSNDSLVSMLNNLVVPAVRSHEVPVRERGLVCLGLCSLLDR~SLAEENLTLFMT
NCU06216.1	NVAGNLQRNDHLVSMLNNLVVPAVRSHEAPVRERGLVCLGLCSLLDR~SLAEENLTLFMH
FG02510.1 	HVSSNLQDNTDLVAMLNNLVVPAVRSHEAPVRERGLVCLGLCALLDR~SLADENLSLFMH
AN8056.1  	NVEGNLQANMNLVTMLNNLVVPAVRSHEAPIRERGLECLGLCCLLDK0TLAEENMTLFIH
          	:* ..** *  **:**************.*:***** *****.***: :**:**::**: 

MG08841.1 	FFSKGHAELQITSLHILTDILNVHGAQLLSANPA-------------LQKVYVKALRGAG
NCU06216.1	FFAKGHTALQITALHILTDILNVHGAQLLSNNTN-------------LLKVYVKALRAGG
FG02510.1 	FFSKGHTALQITALHILTDILNVHGAQLLSSTPG-------------LLKVYVKAVKGGN
AN8056.1  	CYSKGHENLQVTAIHILCDMLTSHPSLLAPVTQADKETVAPPAFQKPLLKVFSRALKP-N
          	 ::***  **:*::*** *:*. * : * . .  ...: :..: ...* **: :*::  .

MG08841.1 	KSPEVQAAATIAVSKLLLGRVVT-------------DPEASAELLKTLVVAYFEPSSSGN
NCU06216.1	RSPEVQAAATVAVSKLLLGRVVS-------------DVDVSAELLKTLVIAYFEPASAEN
FG02510.1 	KAPEVQAAATVAASKLLLGRIVS-------------EEEACQDLLKSLVVAYFDPASSHN
AN8056.1  	SPASVQTAAATALSKLLLTGVFTPSAANIPDAIQEFNQHAIETLLQSLVVSFFHPRTREN
          	 ...**:**: * *****  :.:.:::. ..: .. : ..   **::**:::*.* :  *

MG08841.1 	QSVRQALNYFLPVFCYSRAENQDLMRTVALDALHTLFN------VREGYDDDEADIGDEM
NCU06216.1	QSVRQALNYFLPVFCYSRPENQDLMRRVALDALHTLYN------VREGLDDEEADVDDQM
FG02510.1 	QTVRQALNYFLPVFCFSRAENQELMRSIALDAIHALFN------VRESLEDDDVDVDEDM
AN8056.1  	PALRQALAYFFPVYCHSRPDNTQHMRKITVPVIRTILNSAEEYYSLEAEEDSDGDIDESV
          	 ::**** **:**:*.**.:* : ** ::: .:::: *::..    *. :*.: *:.:.:

MG08841.1 	VS------MSTIGACLVDWTDPRKCYS--AGIPG--DAEKKAVSGDIHLDFACDILAKLG
NCU06216.1	VS------MSTIAACLVDWTDPRKCYNPDEGFSAGLDPEKKTVSADVHLDFAKDILERLQ
FG02510.1 	VS------MTTIGACLVDWTDPRKVFSP----TDLLETEKKAVNGDVHLDLAMDILEKLQ
AN8056.1  	GEKELKALMSGVLGMLAEWTDERRVIGLGGERVLAGGPASSNVCGIIHLQLIKDILERVL
          	 ... .: *: : . *.:*** *:  . .        . .. * . :**::  *** :: 

MG08841.1 	SSI------SK1EEKKVMAALLGKLYVSPTSAED--------------------------
NCU06216.1	GNG------AK1EEKRIMAALLAKLYIPSGSSSSNPSSPI--------------------
FG02510.1 	GNI------TR1EEKKILASLLGKLYVSPGSSKE--------------------------
AN8056.1  	GISEGSNRCSK~EERKLLFSLMSKLYIAPPTAPSRSASQAPEDDSFRSSVRSSHGELNPE
          	.  ..:. .:: **:::: :*:.***:.. :: . .::  ....:  ::  :: .. ...

MG08841.1 	---KIRETYAQMSEAVENQLVSDTTGRNALYKIHVALGKIVNNLDQQQQQMEG-------
NCU06216.1	---KIRDVYALVTNAVENNLLSDATSRNALYKVHVSLGKIVNALDAAAEQDAANNAAGRH
FG02510.1 	---KLREAYQEVSIAVEEGLLSDATSRNQLYKIHVGLGKIVNALDEQQQ-----------
AN8056.1  	NLALAQEVKELLDQTIEEGVAADAASRNALVKVKNVVLKLLAAPMRPSS-----------
          	. :  ::.   :  ::*: : :*::.** * *::  : *::       .           

MG08841.1 	--------YGRRSMSRSTSVGLEDR-TVVP------------------------------
NCU06216.1	ASIASGATTHRRSASRAGSVAPSRAGSVAPPASEASGGGGGGAVSRAGSVAPSASGATAA
FG02510.1 	--------PNYRRASRSTSILTDRQTTEG-------------------------------
AN8056.1  	--------ARGRESSVESDIGSVRSSRSVRPSVEPG------------------------
          	           *  *   .:          .: ...                        

MG08841.1 	-------EDKTITMDTEIKEEEE-------------------------------------
NCU06216.1	PVNSRKRKEREVSVGVSIKEEDENEDEGEDVEMGGTQVGDNDNAGDRETTPKPTATEAQE
FG02510.1 	--------DTSITDEPSIKEEDE-------------------------------------
AN8056.1  	------FGRRGVSVEPSIMEEDE-------------------------------------
          	           ::   .* **:*                                     

MG08841.1 	------------------------------------------------------------
NCU06216.1	PSTEQRRLLRGVSLRSRRQSSQSVVSVQPQQQQEEEQEEQQQQQQPTGRRGSRRASAMKA
FG02510.1 	------------------------------------------------------------
AN8056.1  	------------------------------------------------------------
          	                                                            

MG08841.1 	----------------------EGSDEGTVVHRAETESFTEGETEL--------------
NCU06216.1	KARLSAGRSSRAGTEDIAEEILDDSDGDTVMERTKIEEEDEEEQEEKKREDEEQDDEVGG
FG02510.1 	-----------------------DSD-GTVVPKEDRESLVDSLLSD--GDDVEMTDV---
AN8056.1  	----------------------NEDSRATLDSRMTVIKEEDADAMEE-------------
          	                      . ..  *:  :    .  :     .             

MG08841.1 	--------------------------------------------
NCU06216.1	EEDGKEEEEEEEEEDGDDDDGENEGKDDDELVDELLSDEEDDFE
FG02510.1 	--------------------------------------------
AN8056.1  	--------------------------------------------
          	
```
